# A clinical "near miss" highlights risk management issues surrounding ultrasound-guided and wire-localised breast resections

**DOI:** 10.1186/1754-9493-6-15

**Published:** 2012-07-16

**Authors:** Daniel Richard Leff, Charles Vincent, Ragheed Al-Mufti, Deborah Cunningham, Ara Darzi, Dimitri J Hadjiminas

**Affiliations:** 1 Centre for Patient Safety and Service Quality (CPSSQ), Department of Bio surgery and Surgical Technology, Imperial College London, London, United Kingdom; 2 Department of Breast Surgery, Imperial College Healthcare NHS Trust, London, United Kingdom; 3 Department of Radiology, Imperial College Healthcare NHS Trust, London, United Kingdom

**Keywords:** Breast, Cancer, Ultrasound, Mammography, Wire

## Abstract

**Background:**

The introduction of the National Health Service (NHS) Breast Screening Programme has led to a considerable increase in the detection of impalpable breast cancer. Patients with impalpable breast cancer typically undergo oncological resection facilitated either by the insertion of guide wires placed stereo-tactically or through ultra-sound guided skin markings to delineate the extent of a lesion. The need for radiological interventions on the day of surgery adds complexity and introduces the risk that a patient may accidentally transferred to the operating room directly without the image guidance procedure.

**Case report:**

A case is described of a patient who required a pre-operative ultrasound scan in order to localise an impalpable breast cancer but who was accidentally taken directly to the operating theatre (OR) and anaesthetised without pre-operative intervention. The radiologist was called to the OR and an on-table ultrasound was performed without further consequence.

**Conclusion:**

It is evident that breast cancer patients undergoing image-guided resection are exposed to an additional layer of clinical risks. These risks are not offset by the World Health Organisation surgical safety checklist in its present guise. Here, we review a number of simple and inexpensive changes to the system that may improve the safety of the breast cancer patient undergoing surgery.

## Background

The National Health Service (NHS) Breast Screening Programme led to the detection of over 17,000 new breast cancers between 2009–2010 [1]. Approximately 50 % of surgically treated screen detected cancers had an invasive tumour diameter of less than 15 mm^1^. Impalpable breast cancers require radiological localisation to guide the surgeon as to the approximate location of the lesion within the breast and to help determine the extent of the oncological resection. Localisation is commonly achieved through the use of either guide wires placed stereo-tactically or skin markings under ultrasound guidance. The necessity for pre-operative image-guided interventions introduces additional challenges for the patient (e.g. anxiety, additional psychological morbidity, physical pain associate with wire insertion, etc); the surgeon (i.e. planning, scheduling) and surgical teams (e.g. confirmation that all the necessary pre-operative procedures have been conducted).

Surgeons and surgical teams have of course always been safety conscious. However, systematic studies of error and harm in healthcare have revealed high levels of adverse events, many of which concern surgery [2-4]. In recent years studies of process failures, communication, teamwork, interruptions and distractions have now identified multiple vulnerabilities implicit within systems of surgical care [5-8]. We consider that the case discussed here exposes a number of important weaknesses in the process of care of breast cancer patients undergoing surgery [9].

### Case presentation

**Figure 1:**
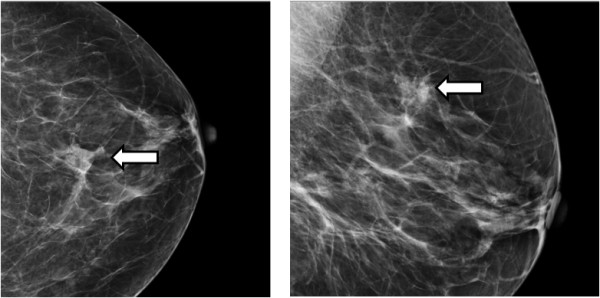
Craniocaudal (CC – left panel) and Mediolateral (MLO – right panel) mammographic views illustrating the suspicious density and pathological microcalcification in the upper outer quadrant of the left breast (white arrows).

A 59-year old Afrocaribbean lady was recently referred to our hospital following confirmation of screen detected left breast cancer. As the lesion was only just palpable, the patient had undergone an image guided biopsy of the radiological abnormality at the host screening centre. Histological assessment of the breast biopsy confirmed invasive ductal carcinoma (IDC). The patient’s case was discussed at a multidisciplinary team (MDT) meeting with review of breast imaging which confirmed the presence of a highly suspicious density in the upper outer quadrant of the left breast (Figure 1), compatible with the site of the known IDC. The agreed plan was for the patient to undergo a wide local excision of the left breast lesion and sentinel lymph node biopsy. Moreover, it was agreed that she would also require a pre-operative ultrasound-guided skin marking on the morning of surgery in order to guide the extent of the breast resection.

She arrived on the morning of her operation and as per hospital protocol was taken to the admissions area for routine checks and observations by the nursing staff as well as to facilitate the consenting process by the surgical team. She was the first patient on the afternoon operating list. The surgical registrar went to see the patient, confirmed the clinical history, imaging findings, histopathological results and MDT recommendations. The patient was informed of the risks and benefits of the proposed procedure as well as the need for image-guided skin marking to assist resectional surgery and it was explained that this would occur prior to her being transferred to the operating theatre. The patient appeared to understand the process, the risks and benefits and duly signed the consent form. The registrar went to the breast imaging department to ensure that the radiologists were expecting the patient for an ultrasound scan. Prior to leaving for the imaging department the registrar explained to the sister in charge of the ‘admissions unit’ that the patient needed to have a pre-operative ultrasound scan and she was not to be transferred to the anaesthetic room until the image guided-skin marking had been conducted. The responsible consultant surgeon, who was otherwise engaged in clinic communicating a new diagnosis of breast cancer to another patient, received a telephone call from the theatre staff informing him that the patient was anaesthetised and enquiring as to his likely arrival time in the operating theatre. No ultrasound skin marking had been conducted. No-one had contacted the surgical team to ask them if it was acceptable to transfer the patient to the anaesthetic room. The patient herself had not queried being transferred to the operating theatre without a pre-operative procedure that only a few minutes beforehand she was informed was an absolute necessity.

The consultant radiologist due to perform the pre-operative ultrasound scan was called to the operating theatre, and thankfully was able to perform the image-guided localisation ‘on-table’ without further insult. The patient subsequently underwent the planned procedure and histopathological assessment confirmed a completely excised 5.2 mm IDC (closest margin 5.5 mm).

## Discussion

**Figure 2:**
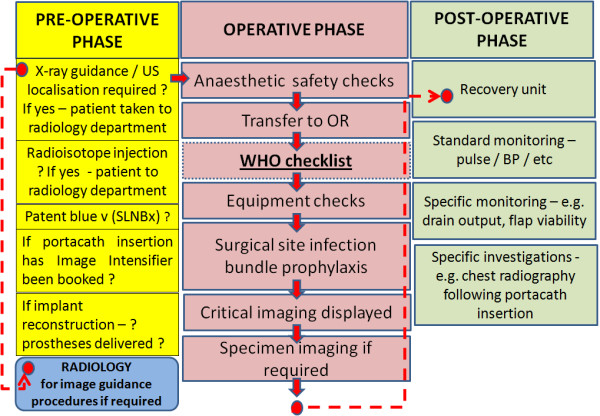
**Flow diagram illustrating the perioperative flow of patient care.** Diagram to illustrate mandatory perioperative safety checks and the flow of the breast cancer patient (depicted as a red circle) through the peri-operative process. Three separate phases of care are illustrated (i.e. pre-operative, operative and post-operative). Safety checks important to each phase of care are highlighted (square box). In the pre-operative phase, the patient requiring image guidance typically has to be taken to radiology in another location within the hospital and then returned to theatre. The patient should only be able to transition phases of care (red –hashed line) when all safety checks have been conducted.

The “near-miss” described here raises several important clinical risk issues surrounding breast cancer patients who require pre-operative procedures prior to cancer surgery. These patients follow a complex pathway with a number of different decision points (Figure 2). The pathways constrain a number of mandatory checks, such as the World Health Organisation (WHO) surgical safety checklist [10], that are integral to the safety of the breast cancer patient undergoing surgery. However, the ‘near miss’ highlights a number of intrinsic vulnerabilities in the system with respect to the flow of the breast cancer patient through the perioperative process. The weaknesses inherent in the system can be summarised as follows:

• ***Asymmetry in safety checks between the pre-operative and operative phase of care.*** As the current case and Figure 2 illustrate, there is a comparative asymmetry in terms of safety checks, with a greater emphasis placed prior to, rather than during the operative phase of care. The WHO surgical safety checklist [10] would not have detected nor prevented the current “near miss” as typically the checklist is conducted *after* the induction of general anaesthesia. There is a clear necessity to formalise the safety briefing *prior to* induction of general anaesthesia. This is essential, not only to ensure that image-guidance has been performed (if required) but that other mandatory pre-operative manoeuvres have been conducted (e.g. radioisotope injection for sentinel lymph node biopsy). Formalised pre-operative safety checks would also act as a vehicle to confirm the surgery side, accuracy of skin markings and to check the availability of allied equipment (e.g. breast prosthesis for post-mastectomy reconstruction).

• ***Issues pertaining to patient proximity and preoperative location***. The current case highlights the potential dangers involved if all patients (whether image guidance procedures are required or not) are observed pre-operatively in the same location. Clearly the flow of patient transport in this situation will vary. Some patient will move directly from the admissions area to theatre and some will be transported to the radiology department first and then subsequently back to the admissions area prior to being taken to the anaesthetic room. This is akin to travellers all arriving at an given airport gate ‘x’ with some people departing for destination ‘z’ and others destined for ‘z’ via another location ‘y’. In such a circumstance there would be a significant chance of boarding the wrong flight. Finally, the proximity of the admission lounge to the operating theatre, which in the case of our NHS Trust is only a few yards, may compound the problem as it may prevent a clear distinction between the two departments in the minds of the healthcare staff. There is therefore always a risk of a patient entering an incorrect pathway. The only factors preventing a patient being incorrectly taken straight to the operating theatre are a member of the surgical team staying with the patient (often impractical); communication between surgeon and admissions / theatre team (failed in the current case); the theatre ‘scrub team’ interpreting the theatre list (should state ‘wire guidance’ or ‘ultrasound skin mark’ as was clearly documented in the current case) and /or the astuteness of the patient who queries being taken straight to theatre, bypassing the radiology department.

• ***Procedural risks specific to the breast cancer patient.*** It is valuable to reflect more broadly on the specific risks to breast cancer patients that are illuminated by the current case. Evidently, not all breast cancer patients are the same and nor are all breast cancer surgeries. Some patients require pre-operative image guidance interventions, some require pre-operative isotope and/or patent blue V injections, others undergoing reconstruction require the availability of a specific breast prosthesis or specialist lipomodelling equipment, whilst certain patients requiring nothing but ‘standard’ operating equipment universal to most operating theatres in the Western world. Those that require pre-operative interventions or specific operative equipment are potentially exposed to another layer of risks that are inherent in the system. The current case highlights the need to make these distinctions, a process that may be aided by changing the emphasis towards safety checks conducted in the pre-operative phase of care (prior to induction) for breast cancer patients.

• ***The importance of pre-operative safety briefing.*** Communication between surgical teams is critical to patient safety. In particular, prior to starting an operating theatre list it is the duty of the surgeon to communicate with the anaesthetist and theatre teams to ensure that all members are aware of the patients being operated on and so that any unique or specific steps can be addressed. Regarding the current case, the briefing was succinct and focused (i.e. list order, patients to be operated on and operation type) and may have dealt better with broader issue surrounding the importance of image guidance, medical past history, and in which patient lesions were deemed to be impalpable. Broader preoperative safety briefings such as this have been shown to improve communication and reduce adverse events [11,12]. In addition to the preoperative briefing, the theatre staff typically inform the surgeon that the patient is about to be transferred to the operating theatre. This is commonly referred to as ‘sending’ for the patient. The theatre staff did not inform the operating surgeon that they were ‘sending’ for the patient. One may argue that the theatre co-ordinator and anaesthetic team should have informed the surgical team that they had summoned the patient to operating theatre. This may have revealed that image-guidance had not yet taken place, thereby circumventing the “near-miss”.

• ***Inability to rely on the patient to prevent errors in flow.*** Surgery is extremely stressful for the patient and they are likely to be anxious, nervous and prone to forgetting information given to them on the day of surgery. While patients should of course be encouraged to speak up if they notice any inconsistencies in their care it is not ultimately their responsibility to monitor the safety of the process. As highlighted here, patients’ may not challenge healthcare staff as to the appropriateness of the procedures they are undergoing. Had the patient informed the theatre staff that she was due to have an ultrasound scan then this “near miss” may not have occurred. Clearly, one cannot rely on the patient to prevent such an incident, and a new process of safety checks for breast cancer patients may be required. Of great concern is that a breast patient can currently arrive in the operating without a series of systematic safety checks being performed prior to the induction of general anaesthesia.

## Conclusions

What should be done to enhance patient safety? ‘Naturally, individual centres need to implement their own specific solutions to minimise risks to patients during the care pathway. However, in our centre we are exploring the following fundamental processes changes that may help to enhance patient safety and prevent similar adverse events:

1. Breast cancer patients requiring pre-operative localisation should not enter the operating theatre space or admissions area until the wire-insertion and / or image-guided skin marking have been conducted. These patients should go directly to the radiology department on the morning of surgery.

2. Patients should not be consented by the surgical team until the necessary localisation procedures have occurred. This may be complicated by the fact that pre-operative consent for wire-guided excision is, in effect used as consent for the wire-localisation procedure as well as the subsequent wide local excision. Nevertheless, the absence of a signed consent form typically acts a barrier to proceeding with induction of general anaesthesia and would have circumvented the current adverse event.

These strategies may reduce the risk to breast cancer patients in our NHS Trust but are not guaranteed to have the same effect within the wider NHS and beyond. Ultimately, an adequate pre-operative safety briefing along with specialty specific modifications and an adherence to the WHO checklist [13,14] are more likely to lead to a consistent and sustainable improvement in patient safety. Regarding the latter, the WHO surgical safety checklist should be adapted for the needs of the breast cancer patient, along the lines illustrated in Table 1. It is worth noting that adaptations to the checklist are not novel and similar adjustments to those proposed have been made for cataract surgery [15]. Clearly, a breast cancer specific checklist would place greater emphasis on checks in the pre-operative phase including the need for pre-operative localisation procedures, radioisotope injections, availability of gamma probes and patent blue V injectate. Critically, in our view these checks should take place prior to induction of general anaesthesia.

**Table 1:** Proposed variation on the WHO surgical safety checklist to augment the pre-operative safety briefing and enhance the safety of the breast cancer patient undergoing surgery

			
Read aloud before administering general anaesthesia			
Has the patient confirmed their name / site of surgery / procedure and consent?	YES	NO	
Is the anaesthetic machine / medications complete?	YES	NO	
Does the patient have an allergy (especially to blue dye)?	YES (if yes – details)	NO	
Which breast / axilla is being operated on?	Right	Left	Both
Is the surgical site marked?	YES	NO (If ‘NO’ - do not administer GA, consult with surgeon / surgical team)	
Is a pre-operative localisation procedure required?	YES	NO	
If a pre-operative localisation procedure is required, has it been performed?	YES	NO (If ‘NO’ - do not administer GA, consult surgeon / surgical team)	N/A
Is a SLNB procedure being conducted?	YES	NO	
If a SLNB procedure is being conducted has radioisotope injection been administered and is patent blue V / gamma probe available?	YES	NO (If ‘NO’ - do not administer GA, consult surgeon / surgical team)	N/A
Is the patient having a breast prosthesis inserted?	YES	NO	
If a breast prosthesis is required has it been ordered / available in theatre?	YES	NO (If ‘NO’ - do not administer GA, consult surgeon / surgical team)	N/A

Thankfully, the current patient came to no actual harm as a result of the “near miss” but should this adverse event have occurred in a patient requiring stereotaxic guidance for wire positioning, reversal of the general anaesthetic and transfer to the x-ray department would have been mandated. In light of this case we are exploring the pre-operative patient pathway for breast cancer surgery in our own Trust and are lobbying the WHO surgical safety team to develop a more specific checklist to maximise the safety of the breast cancer patient undergoing surgical interventions within the National Health Service.

## Competing interests

The authors state that they have no conflict of interest to declare.

## Authors’ contributions

The patient was treated by DRL, DC, RAM, and DH, data was prepared and reviewed by DRL, CV, DC and DH. The manuscript was drafted by DRL, CV and DH and critically edited by AD. The discussion and recommendations enclosed were discussed between DRL, DC, RAM, AD and DH. All authors read and approved the final manuscript.

## References

[B1] NHS National Screening Programme and Association of Breast Surgery An audit of screen detected breast cancers for the year of screening April 2009 to March 2010 2010 West Midlands: West Midlands Cancer Intelligence Unit

[B2] Kable AK Gibberd RW Spigelman AD Adverse events in surgical patients in Australia Int J Qual Health Care 2002 14 269 76 10.1093/intqhc/14.4.269 12201185

[B3] Gawande AA Thomas EJ Zinner MJ Brennan TA The incidence and nature of surgical adverse events in Colorado and Utah in 1992 Surgery 1999 126 66 75 10.1067/msy.1999.98664 10418594

[B4] Vincent C Moorthy K Sarker SK Chang A Darzi AW Systems approaches to surgical quality and safety: from concept to measurement Ann Surg 2004 239 475 82 10.1097/01.sla.0000118753.22830.41 15024308 PMC1356252

[B5] Healey AN Sevdalis N Vincent CA Measuring intra-operative interference from distraction and interruption observed in the operating theatre Ergonomics 2006 49 589 604 10.1080/00140130600568899 16717011

[B6] Undre S Sevdalis N Healey AN Darzi SA Vincent CA Teamwork in the operating theatre: cohesion or confusion? J Eval Clin Pract 2006 12 182 9 10.1111/j.1365-2753.2006.00614.x 16579827

[B7] Nagpal K Vats A Lamb B Ashrafian H Sevdalis N Vincent C Moorthy K Information transfer and communication in surgery: a systematic review Ann Surg 2010 252 225 39 10.1097/SLA.0b013e3181e495c2 20647929

[B8] Williams RG Silverman R Schwind C Fortune JB Sutyak J Horvath KD Van Eaton EG Azzie G Potts JR 3rd Boehler M Dunnington GL Surgeon information transfer and communication: factors affecting quality and efficiency of inpatient care Ann Surg 2007 245 159 69 10.1097/01.sla.0000242709.28760.56 17245166 PMC1877003

[B9] Vincent CA Analysis of clinical incidents: a window on the system not a search for root causes Qual Saf Health Care 2004 13 242 3 10.1136/qshc.2004.010454 15289620 PMC1743862

[B10] National Patient Safety Agency WHO surgical safety checklisthttp://www.nrls.npsa.nhs.uk/resources/clinical-specialty/surgery/ [accessed 28th January, 2012]

[B11] Einav Y Gopher D Kara I Preoperative briefing in the operating room: shared cognition, teamwork, and patient safety Chest 2010 137 443 9 10.1378/chest.08-1732 20133291

[B12] Lingard L Regehr G Orser B Reznick R Baker GR Doran D Espin S Bohnen J Whyte S Evaluation of a preoperative checklist and team briefing among surgeons, nurses, and anesthesiologists to reduce failures in communication Arch Surg 2008 143 12 7 discussion 8 10.1001/archsurg.2007.21 18209148

[B13] van Klei WA Hoff RG van Aarnhem EE Simmermacher RK Regli LP Kappen TH van Wolfswinkel L Kalkman CJ Buhre WF Peelen LM Effects of the introduction of the WHO "Surgical Safety Checklist" on in-hospital mortality: a cohort study Ann Surg 2012 255 44 9 10.1097/SLA.0b013e31823779ae 22123159

[B14] Truran P Critchley RJ Gilliam A Does using the WHO surgical checklist improve compliance to venous thromboembolism prophylaxis guidelines? Surgeon 2011 9 309 11 10.1016/j.surge.2010.11.024 22041642

[B15] National Patient Safety Agency WHO surgical safety checklist: for cataract surgery onlyhttp://www.nrls.npsa.nhs.uk/resources/clinicalspecialty/surgery/ [accessed 28th January, 2012]

